# Ethical Dilemmas in Disaster Medicine

**Published:** 2012-10-30

**Authors:** C Ozge Karadag, A Kerim Hakan

**Affiliations:** 1 Department of Public Health, Faculty of Medicine, Hacettepe University, Ankara, Turkey; 2 Department of Public Health, Faculty of Medicine and Disaster Research and Implementation Center, Hacettepe University, Ankara, Turkey

**Keywords:** Disasters, Ethics, Bioethics, Disaster medicine, Public health

## Abstract

**Background:**

Disasters may lead to ethical challenges that are different from usual medical practices. In addition, disaster situations are related with public health ethics more than medical ethics, and accordingly may require stronger effort to achieve a balance between individual and collective rights. This paper aims to review some ethical dilemmas that arise in disasters and mainly focuses on health services. Disasters vary considerably with respect to their time, place and extent; therefore, ethical questions may not always have `one-size-fits-all` answers. On the other hand, embedding ethical values and principles in every aspect of health-care is of vital importance. Reviewing legal and organizational regulations, developing health-care related guidelines, and disaster recovery plans, establishing on-call ethics committees as well as adequate in-service training of health-care workers for ethical competence are among the most critical steps. It is only by making efforts before disasters, that ethical challenges can be minimized in disaster responses.

## Introduction

The word “ethics” takes its roots from the Greek term “ethikos” or “ethike” from “ethos”, which can be translated as morals.(1) Ethics is the study of standards of conduct and moral judgement, or a system or code of morals.(2) Modern bioethics is founded on four basic principles; which are the principles of beneficence, non-maleficence, respect for autonomy, and justice.(3) The priority of these principles may change with different circumstances,(4) such as in disasters, which sometimes may lead to challenges that are quite different from usual medical practices. In addition, disaster situations are related with public health ethics more than they do with medical ethics, and accordingly may require stronger effort to achieve a balance between individual and collective rights. In public health, there is generally a conflict between autonomy of the individual and the desire to protect and promote the health of the population.(3) This “dual loyalty”, which also exists in many disaster situations, stands in the middle of most ethical dilemmas. Based on these matters, our paper aims to review some of the ethical dilemmas that arise in the context of disasters and mainly focuses on health services in disaster management.

## Ethics in Disaster Medicine

Ethics in disaster medicine deals with the ethical issues and dilemmas in natural and human-made disasters. Disaster ethics is a very broad field, in a sense that it compasses numerous topics from individual to collective ethics; nevertheless, it focuses mainly on macro-ethics rather than micro-ethics. Disaster ethics is usually addressed in three phases: (i) pre-disaster (pre-event) or preventive phase, ii) disaster (event/ crisis) and early response phase, and iii) post-disaster (post-event) or rehabilitation phase.(1,4)

Although each phase may pose different ethical dilemmas, the main topics of interest in these phases can be summarized as follows: preventive ethics, disaster triage, informed consent, communicable disease surveillance, risk communication, quarantine/ isolation, vaccinations, refusal of medical treatment, euthanasia, allocation of resources, linguistic, religious, and cultural differences, vulnerable groups, community participation, division of labour, healthcare workers` duty to treat, obligations to disaster relief workers, participation of health-care workers in war crimes/torture/death penalty, relations with industry and media, disaster response and development, and disaster research. It is worth noting that, in spite of this wide range of ethical issues in disasters, codes of ethics that are specific for disasters remain to be scarce around the world.(4)

## Ethics in Pre-Disaster Phase

Today, developing strategies to prevent disasters or to decrease the magnitude of the disaster related injuries and damages is regarded as an ethical responsibility.(5) Developing a preventive ethics approach in this pre-disaster phase, also helps to reduce conflicts during the crisis phase.(6) Within this scope, capacity building to increase knowledge and skills of disaster relief professionals and the populations at risk, developing disaster recovery plans, practicing and updating these plans as needed, building strong partnerships among organizations and institutions with potential duties in disaster relief, preparing legislations and manuals as to better respond to the ethical conflicts in disasters as well as informing all partners about this ethical framework are crucial.(6,7) Accordingly, the World Medical Association (WMA) recommends that disaster medicine training be included in the curricula of university and post-graduate courses in medicine.(8)

## Ethics in Disaster and Early Response Phase

Main ethical principles in the provision of health services during event and early response phase of disasters are the principles of non-maleficence, beneficence, justice, and the respect for autonomy. In this phase, reaching the disaster site as quickly as possible is the most crucial step. In line with the principles of the ethical practice of public health, “Public health institutions should act in a timely manner on the information they have within the resources and the mandate given to them by the public”.(9) If the health authorities and health-care workers act slowly, ignoring the fact that time is vital, they may be late in saving lives and violate the principle of doing no harm.(10)

Triage, as the second most important step, is often considered critical in the distribution of limited medical resources, where highest priority should be given to the principles of beneficence and justice.(11) According to the WMA Statement on Medical Ethics in the Event of Disasters (1994); “In selecting the patients who may be saved, the physician should consider only their medical status, and should exclude any other consideration based on non-medical criteria”.(8) Ongoing discussions on triage decisions with respect to the victim`s age, gender, social status, ethnic origin or profession (e.g. health workers) also conflict with the basic right to live at the individual level and justice principle, in general. In their discussion, Halpern and Larkin (2006) conclude that “Ideological issues must not eclipse the humanistic priorities embodied in ethical rules”.(4)

Until today, many systems (e.g. START, SIEVE, Homebush Triage etc.), have been proposed for the so-called primary triage or scene triage, which is defined as the initial assessment of victims at the disaster site.(4,12)

WMA recommends that the physicians should set an order of priorities for treatment that will save the greatest number of lives and restrict morbidity to a minimum. In connection with this, some patients, whose condition exceed the available resources, may be classified as "beyond emergency care". According to WMA (1994), “It is ethical for a physician not to persist, in treating individuals "beyond emergency care". However, the physician must show such patients compassion and respect for their dignity, for example by separating them from others and administering appropriate pain relief and sedatives”.(8) Halpern and Larkin (2006) contribute to this discussion by stating that health care has to be equitably distributed rather than equally, with each victim receiving care according to medical need.(4) On the other hand, critical questions on when and how to apply disaster triage still remain. Domres et al. (2001) argue that disaster triage is ethical only under extreme situations.(13) Sztajnkrycer et al. (2006) also contribute to this discussion by reporting that resources are rarely scarce in events viewed by the general public as disasters and the number of affected people may be misleading to switch to disaster triage. The authors argue that the current disaster triage, which is based on the concept of utilitarianism, aims to maximize benefit of the society, at the expense of individual needs, which may not be acceptable in modern society.(12)

In literature, there are numerous studies that reveal high rate of overtriage and undertriage in disaster situations.(4) Studies on triage decisions using tabletop exercises also show that triage decisions of health professionals vary to a great extent,(12,14) which indicate that training and experience of health professionals with respect to disaster triage is crucial to have medically and ethically sound decisions. In accordance with the literature findings, WMA recommends that disaster triage should be entrusted to authorized, experienced physicians, assisted by a competent staff.(8)

Informed consent, which is used frequently on daily medical practice, is another important ethical challenge in disasters. The WMA Declaration of Lisbon on the Rights of the Patient (1981) states that “If the patient is unconscious or otherwise unable to express his/her will, informed consent must be obtained, whenever possible, from a legally entitled representative. If a legally entitled representative is not available, but a medical intervention is urgently needed, consent of the patient may be presumed, unless it is obvious and beyond any doubt on the basis of the patient’s previous firm expression or conviction that he/she would refuse consent to the intervention in that situation”.(15,16) On the other hand, there might be exceptions to informed consent, such as in disaster and other public health emergency situations.(11) WMA Statement on Medical Ethics in the Event of Disasters (1994) states that in a disaster response, it should be recognized that there may not be enough time for informed consent to be a realistic possibility.(8) Although health professionals in disaster relief are expected to make every effort to start and sustain available treatments according to priority, some victims might refuse treatment. In that situation, mental health state of the victim should be assessed. If there is any doubt, the treatment should be continued to avoid any medical or legal consequences. If the examination reveals no significant mental problem, then health professionals may try to convince the person for the recommended treatment, whenever possible.(4) If time permits, the last option might be to ask the victim to sign a document indicating that s/he does not accept the treatment.(10)

Just as in daily medical practice, there may be circumstances in disasters that some severely injured patients ask for euthanasia. It should be emphasized that today, euthanasia is prohibited in public international laws as well as most medical codes of ethics around the world.(1) This prohibition has also been restated by the WMA in its Declaration on Euthanasia (1987), which states that the act is unethical.”(17)

On the other hand, competent patients have the right to refuse any medical treatment, even if the refusal results in their death. Once physicians have made every effort to provide patients with information about the available treatments and their likelihood of success, they must respect the patients’ decisions”.(16)

Refusal of treatment might be more complex in pandemic disasters. Although refusal of treatment usually seems as an individual decision, the patient`s right to refuse treatment may conflict with the health professionals` duty to protect public health. Patients with highly contagious diseases may pose a significant threat for other people. In these situations, every effort should be made for the diagnosis and proper treatment of infected persons, including trying to convince the patients, who refuse treatment, by explaining the potential health risks for public health.(18)

Pandemic disasters may also pose other ethical dilemmas with respect to the autonomy of individuals. Although the WMA International Code of Medical Ethics (1949) states that “A physician shall owe his/her patients complete loyalty”,(19) it is generally accepted that physicians may in exceptional situations have to place the interests of others above those of the patient. Mandatory reporting of patients who suffer from designated diseases is one such exception. According to WMA, physicians should fulfil their duty to report, although patients should be informed that such reporting will take place.(16) When meeting public health requirements, every effort should be made to minimize any harm to individuals. Securing patients` identity information during reporting will both help to ensure privacy and protect individuals from infection-related stigma and discrimination.

Public health measures in pandemic disasters, such as vaccination campaigns, risk communication, quarantine and isolation are also worth noting with respect to potential ethical dilemmas. In all of these dual-loyalty situations, protection of public from harm is usually regarded as a superior goal than respect for autonomy. In the case of vaccination, it is widely accepted that risk-benefit ratios must be calculated for all immunizing agents.(3) Although vaccines can carry some health risks, the risk of harm from vaccines are much less than the risks of morbidity and mortality from infectious diseases.(20) For the very reason, the ethical approach should be to prioritise the common good, but also to inform the public about potential benefits and risks before beginning mass vaccinations in disaster situations.

According to Last (2004), scientists working in emergency situations like an epidemic, have an ethical duty to be open in dealing with the public. Last argues that public has the right to know what the experts know. Within this scope, implementing the principles of risk communication to avoid unnecessary fear and anxiety among the public is of vital importance. One of the ethical issues that arises in risk communication is the risk of stigmatization in certain sub-groups.(20) The ethical approach should be to minimize generalizations about high risk groups, whenever possible.

In disaster situations, delivery of appropriate and updated information to health-care workers on a regular basis is also critical to minimize misinformation, mistrust and refusal of public health measures among the public. Joint Centre for Bioethics (JCB) Pandemic Influenza Working Group at the University of Toronto (2005) reports that “If ethics are clearly built into pandemic plans in a transparent manner, the plans carry greater trust, authority and legitimacy”.(6) According to Soliman and Rogge (2002), information helps survivors make informed decisions that are intrinsically related to their life arrangements and future well being.(7)

Pandemic disasters may sometimes require to take stronger measures such as closing schools, imposing isolation and quarantine, which restrict freedom. Identifying and isolating cases for the common good is an accepted feature of communicable disease control.(3) Nevertheless, all existing data should be monited carefully and the risk of stigmatization of isolated individuals or groups should be discussed before taking such measures. Pandemic Influenza Working Group at the University of Toronto (2005) recommends decision makers to make decisions in a fair manner, use the least restrictive method without discrimination, explain the rational and provide support services to the people affected by the restrictive measures. The ethical guide assembled by the Working Group for pandemic influenza includes 10 substantive values, which are; individual liberty, protection of the public from harm, proportionality, privacy, equity, duty to provide care, stewardship, solidarity, trust, and reciprocity. The guide also recommends decisions in pandemic situations to be based on reasons, as well as decision-making processes to be open, transparent, inclusive, responsive and accountable.(6) With a similar goal, the World Health Organization (WHO) developed a guide for influenza pandemic preparedness planning, calling on planners to use an ethical framework in dealing with issues such as quarantines, allocation of resources, and vaccinations.(6) WMA Statement on Medical Ethics in the Event of Disasters (1994), Code of Conduct for the International Red Cross and Red Crescent Movement and Non-Governmental Organizations in Disaster Relief (1995) as well as several UN guidelines were also developed for the same goal of guiding disaster relief efforts within an ethical framework.(8,21)

Allocation of resources, as mentioned in guidelines, also create ethical dilemmas in disasters. WMA Declaration of Lisbon on the Rights of the Patient (1981) points out to the major role of physicians in the allocation of healthcare resources.(15) In this regard, physicians may sometimes have to give priority to the principle of justice over their patient`s autonomy and to avoid delivery of unnecessary health services.(16)

WMA Declaration of Lisbon on the Rights of the Patient (1981) states this role as the following: “In circumstances where a choice must be made between potential patients for a particular treatment that is in limited supply, all such patients are entitled to a fair selection procedure for that treatment. That choice must be based on medical criteria and made without discrimination”.(15,16) Development of clinical practice guidelines in the pre-disaster phase and using these guideline-based criteria in health resource allocation in the response phase may minimize potential ethical conflicts that arise during decision-making in disasters. In this regard, Lin and Anderson-Shaw (2009) proposed a clinical model for decision making in allocation of mechanical ventilators in disaster/pandemic situations, which was based on the ethical principles of beneficience and justice and utilized the concept of triage. In the model, the researchers recommended formation of a pandemic triage committee to allow decisions to be made by a team of professionals rather than individual physicians. In addition, they proposed a palliative care protocol and early family involvement for families of patients to be aware of the protocols, thus avoiding potential conflicts.(22) JCB Pandemic Influenza Working Group at the University of Toronto (2005) recommended that governments and the health care sector should engage public, and other partners in determining the criteria to make resource allocation decisions, and should ensure that rationales for allocation decisions are publicly accessible.(6)

In many devastating disasters, international or non-governmental organizations come to the affected area to give assistance on different fields and to contribute to the overall disaster relief; however, they usually have different mandates and principles of working. International or non-governmental organizations also vary considerably on their ethical values, which motivate them.(2) Likewise, it is expected that countries with different social structures and beliefs have different perspectives on international ethics of assistance.(23)

The division of labor among organization and institutions is considered as one of the ethical aspects of disaster response. Accordingly, every effort should be made to assign labors according to the expertise of each organization. Hussein (2010) contributes to this issue by stating that networking with other service providers is both an ethical and an operational need.(24) Spending available financial resources, as another ethical issue in disaster response, should also be considered. According to the UNDP (1997), millions of dollars are spent on salaries, per diems, transportation, and other costs for the disaster relief experts of countries other than the affected country; however, disaster response spending should primarily be done by contracting services from the affected communities themselves.(2)

Health workforce is one of the most important human resources in disaster response. On the other hand, this workforce might be negatively affected by disaster conditions (e.g. pandemic outbreaks, environmental pollution, military conflicts), which may pose significant threats for the relief workers` own safety and health.(11) Hesitation of health-care workers to perform their duty in pandemic disasters is one such example. Many studies in literature indicate that health professionals constitute a significant proportion of the victims in pandemic situations.(25,26) In addition, numerous literature findings reveal unwillingness of at least some health professionals` to treat patients with communicable diseases.(27,28) In daily practice, medical codes of ethics make no exception for infectious patients with regard to the physician’s duty to treat all patients equally;(16) however, disaster conditions might have its own unique risks. Therefore, the question of where to draw the line between acceptable and unacceptable level of risk for health-care workers still remains to have more concrete answers.

According to a recent WHO publication (2007) on ethical considerations in pandemic influenza; “Countries should develop policies that clearly delineate health-care workers’ obligations, which can be recognized in one or more of the following ways: moral obligations, professional obligations, contractual obligations, non-contractual legal obligations.” Furthermore, it is stated that the duty of health-care workers to work with health risks is not unlimited. The guide concludes that “From an ethical perspective, the least problematic enforcement mechanisms are those that have been voluntarily adopted by those who will be affected by them. Thus, governments should encourage professional organizations to develop policies regarding professionals’ obligations to work during epidemic”.(29) WHO (2007) also recommends governments and employers to minimize risks to health-care workers by giving adequate education and by taking preventive measures, which the health-care workers, by an ethical obligation are expected to comply with. In case of morbidity or mortality of health-care workers, medical and social benefit systems are proposed.(29) Pandemic Influenza Working Group at the University of Toronto (2005) recommends human resource strategies for communicable disease outbreaks to be equitable with respect to the distribution of risk among individuals and occupational categories, as well as disability insurance and death benefit to be available for staff and their families.(6) WMA, in its Statement on Medical Ethics in the Event of Disasters (1994) also calls upon governments and insurance companies to cover civil liability and personal damages to which physicians might be subject in disaster or emergency situations. The WMA also requests governments to accept participation of foreign physicians to relief work without any discrimination.(8) According to the UNDP (1997), relief institutions have special ethical obligations to their staff during humanitarian emergencies. Adequate preparation and training beforehand, and effective counseling and support during and after operations are strongly advised.(2)

In literature, complex humanitarian emergencies and armed conflicts are often classified as disasters. In relation with the duty of health professionals in emergency and disaster situations, participation in the acts of torture, death penalty or inappropriate treatment constitute a critical ethical challange. WMA, in its Resolution on the Responsibility of Physicians in the Denunciation of Acts of Torture or Cruel or Inhuman or Degrading Treatment (2003), states that “Physicians should guard their professional independence to determine the best interests of the patient and should observe, the normal ethical requirements of informed consent and confidentiality. Physicians should report to the appropriate authorities any unjustified interference in the care of their patients, especially if fundamental human rights are being denied. If the authorities are unresponsive, help may be available from a national medical association, the WMA and human rights organizations”.(16,30) WMA, with the Declaration of Hamburg Concerning Support for Medical Doctors Refusing to Participate in, or to Condone, the Use of Torture or Other Forms of Cruel, Inhuman or Degrading Treatment (1997), is responsible to support physicians who resist to be involved in such inhuman procedures or who work to treat and rehabilitate victims.(16)

Respect for diverse values, beliefs, and cultures in the community consitutes one of the principles of the ethical practice of public health.(9) Disasters generally create situations, in which, some health services are delivered by health-care workers who are originally not from the affected area. Foreign health-care workers, whether from the affected country or from another country may have difficulties in communicating with the patients or treating them.(16)

Besides interfering with optimum health care; cultural, religious and linguistic barriers may also have significance with respect to creating ethical dilemmas. If health professionals and patients do not speak the same language, every effort should be made to find interpreters.(16) However, in presence of cultural or religious differences, interpreters may not be enough to overcome communication problems. Without any preparation, international relief workers may be at risk for delivering culturally inappropriate services, such as distribution of condoms to adolescents of a conservative community.(24) Such interventions might negatively affect the overall relief efforts. Therefore, foreign profesionnals have an ethical duty to be aware of any cultural and religious differences, when delivering preventive and curative health services. According to the WMA Statement on Medical Ethics in the Event of Disasters (1994); the physician must respect the customs, rites and religions of the patients.(8) In this respect, community participation in disaster relief efforts is a useful approach in planning services, which are ethically sound and widely accepted by the affected community. Ensuring an opportunity for input from community members is also one of the principles of the ethical practice of public health.(9) This approach helps to deliver services on a needs-based. Most studies in literature indicate that participation of community members make significant changes in the recovery phase of disasters.(2,7,31)

Another principle of the ethical practice of public health is the empowerment of disadvantaged community members.(9) Health professionals working in disaster response should pay special attention to vulnerable groups; including children, women, elderly, people with disability, refugees and other minority groups,^7^ since these groups are usually affected more negatively than the general population.(2) In line with the ethical principle of justice, it is also crucial for relief workers to try avoiding actions that may cause stigmatization and discrimination of vulnerable groups.(3)

Vulnerability is also related with the topic of disaster research, which is among the most important ethical challanges of disaster medicine. Today, there are numerous policy documents, such as WMA Declaration of Helsinki (1964), International Ethical Guidelines for Biomedical Research Involving Human Subjects (2002) and The Ethics of Research Related to Healthcare in Developing Countries (2002) that guide health professionals in their scientific research;(16,32-34) however, disaster situations may pose their own conflicts with respect to the study group, informed consent, etc. That`s why research in disasters may be difficult to perform according to the existing declarations.(23) The event and early response phase of disaster, where there is greatest respondent vulnerability and least social order to conduct research, is known as the “Bermuda Triangle” of disaster research. In long term recovery period, the social order increases, whereas the vulnerability of affected people decreases.(35) According to Abramson (2007), the social context of a disaster defines the research and the ethical landscape. In relation with this context, he proposes researchers to ask several critical questions before conducting any disaster research: timing of the study, presence of any time limit, nature of exposure to disaster or agent, characteristics and vulnerability of the study group, potential risks and benefits to study participants, local support, informed consent, referrals for help, logistics and potential risks to the research team are the main questions that have to be answered by the researchers to plan disaster studies in the most appropriate and ethical way.(35) According to the principles of the ethical practice of public health, public health institutions should protect the confidentiality of information that can bring harm to an individual or community;(9) however, media interest in the disasters and people affected by disasters raises ethical issues on privacy and the principle of respect for autonomy.(36,37)

Media plays an important role in dissemination of information for both the general community and disaster victims. In addition, disasters covered by the media receive more attention;(23) however, media news may interfere with the private life of the victims.(7) In the Code of Conduct for the International Red Cross and Red Crescent Movement and NGOs in Disaster Relief (1995); it is stated that in the information, publicity and advertising activities, the disaster victims should be recognized as dignified humans, but not as hopeless objects.(21) In addition, the WMA Statement on Medical Ethics in the Event of Disasters (1994) states that the physician has a duty to each patient to ensure confidentiality when dealing with third parties.(8) It is also important to designate health-care workers, who are experienced in media relations. Another ethical issue with media relations is that some organizations tend to work in disaster relief primarily for the media coverage, since they link future funding options with their image in the media.(23) Here, the ethically appropriate approach would be to provide assistance with the primary and only goal to help disaster victims, which will eventually be followed by positive responses from both donors and the public in general.

## Ethics in Post-Disaster Phase

All ethical values and principles that were mentioned for pre-disaster and early response phases should also be recognized in the aftermath of disasters; however, health care professionals should act according to the requirements of the new phase. WMA Statement on Medical Ethics in the Event of Disasters (1994) states that “In the post-disaster period, the needs of survivors must be considered. Many may have lost family members and may be suffering psychological distress. The dignity of survivors and their families must be respected”.(8)

According to the UNDP (1997), a disaster response should prevent future disasters and decrease vulnerability of the victims. Opposite to common thinking, developmental efforts should start with the early stages of disaster response, rather than the post-disaster phase. This approach avoids development of a dependency syndrome among the people affected by the disaster. Participation of the community to disaster response does not only help to determine priority needs of and culturally appropriate interventions for the affected community, but also helps to actively engage people to work for their community`s own rehabilitation and development.(2)

Hussein (2010) argues that dependence on foreign aid, directing financial resources to NGO`s rather than the local health infrastructure, shift of the local health-care workers to international NGOs, where they are better paid and inappropriate division of labor, where similar health services are delivered by different relief organizations delay the development process in the affected community. Therefore, starting with the earliest possible time in disaster response, efforts should be made to build the infrastructure of the healthcare system, with the community`s own human resources for health.(24)

## Conclusion

Disasters vary considerably with respect to their time, place and extent; therefore, ethical questions in these situations may not always have `one-size-fits-all` answers. On the other hand, embedding ethical values and principles in every aspect of health-care is of vital importance in disasters. For the very reason; reviewing legal and organizational regulations, developing health-care related guidelines, protocols and disaster recovery plans by taking potential ethical dilemmas into account, establishing on-call ethics committees as well as adequate in-service training of health-care workers for ethical competence are among the most critical steps to take in pre-disaster phase. These measures should be taken both at the local level as well as the country level. In conclusion, it is only by making great efforts before disasters, that ethical challenges can be minimized in disaster responses.
